# Report of Cases of Hospital Gangrene

**Published:** 1864-12

**Authors:** William Thompson

**Affiliations:** Assistant-Surgeon, U.S.A.


### REPORT OF CASES OF HOSPITAL GANGRENE.

By WILLIAM THOMSON, M.D., Asst.-Surgeon, U.S.A.

From the American Journal of the Medical Sciences.

The following histories of cases of hospital gangrene treated in Douglas Hospital, Washington, serve to illustrate several points of great interest in the etiology, pathology, and treatment of a disease hitherto rare in our military hospitals, and most worthy of careful study.

The victims of this disease were wounded at Fredericksburg, Va., Dec. 13th, 1862. For several weeks previous to this battle, the army had been resting on the Rappahannock, and had been exposed to no great hardships. It had been amply supplied with good and varied food, and the men were free from any scorbutic or other cachectic taint.

It is well known that the fullest preparations had been made by Surgeon Letterman for that engagement. The operations were performed promptly, and the wounded probably received better care than ever before in the history of the war.

On the 26th of December, 1862, about two hundred wounded from the battle of Fredericksburg, were received into the Douglas Hospital.

I cannot speak of their treatment, medical, surgical, or hygienic, as at that time I was not connected with the hospital. I am, however, aware that the building in which the gangrene appeared contained fifty badly wounded, and recent cases; that there was a deficiency of medical officers and dressers to insure the necessary cleanliness, and that the sanitary condition of the ward was far from perfect.

This hospital consists of three brick houses known as "Minnesota Row;" and two large wooden pavilions, each divided into two wards.

The ward in which this disease originated is one hundred and forty-three feet in length, twenty-three feet in breadth, and sixteen feet in height (eighteen feet at the cone, and fourteen at the eaves of the roof), and contains beds for fifty patients, thus giving 1050 cubic feet of space for each bed. There are two rows of windows, the lower of which contains two, the upper one sash each; the upper windows are so constructed as to be opened by means of a cord, but when opened directing a current of cold air immediately downwards upon the beds beneath.

On the 16th of Feb., 1863, I took charge of the hospital, and found the ward mentioned in the following condition: There was no ridge ventilation, nor was there any egress for foul air, except through two large wooden shafts connected with two of the stoves, which had been placed there only a few days previously. The ward was heated by ordinary sheet-iron radiating coal stoves; and no provision had been made, until a few days before, to introduce any supply of fresh air. It contained from forty to fifty patients, all wounded—many of them very severely. The police was not unexceptionable, too little attention having been paid to the removal of offensive discharges. The medical officer in charge preferred to water, as a dressing, either simple cerate or mutton tallow, which had been issued to the hospital, and which had become rancid. The attendants were, from a want of strict discipline, careless and inattentive. There was a perceptible and offensive odor in the ward, which felt close and badly ventilated; and this condition of the atmosphere seemed to have a marked effect on the spirits of the men; they were all gloomy, despondent, and homesick.

On Feb. 17th, when making my first visit with the officer in charge of the ward, I discovered

Case I. Sergt. Otto Kosack, Co. K, 2d Md. Vols., who had been struck by a shell, Dec. 13th, 1862, which made it necessary to amputate his left leg at the middle. The operation was done ten minutes after the injury. He was received here on the 26th Dec. The stump closed by granulation, a small portion of the tibia having been removed by exfoliation. The cicatrization had been almost complete, when, a few days previous to the 17th, the still open wound commenced to slough. He was anæmic, very pallid, haggard, and with an expression of great depression in his features; his pulse was very feeble and rapid. He had been "feeling very badly" for several days, and complained of a burning pain in the stump.

On the outer side of the tibia there was an ulcer, one inch in diameter, covered with a yellowish-gray, pultaceous slough, and a serous and very fetid discharge; the edges were thickened and everted, and an areola of purple, livid congestion extended for half an inch from the margin, which was undermined.

This sore was at once treated with pure nitric acid, applied both to the ulcer and to the areola; the ulcer was dressed with an antiseptic solution of creasote; and citrate of iron and quinine, with stimulants and nutrients were freely given.

On the 18th, the sloughing had extended to the border of yesterday's livid areola, but was now more superficial; and the areola, which had likewise invaded the surrounding skin, was more florid. The ulcer was now two inches in diameter. As there was some doubt as to its specific character, the patient was not removed from the ward until the 23d, when he was transferred, with several others, to a small ward prepared in the brick building, and completely isolated from the other wounded men.

The iron and quinine was found to disturb his stomach and destroy his appetite, and was replaced by a mixture of nitro-muriatic acid and tincture of opium.

*March 3d.* The sore was now perfectly healthy, and was granulating rapidly.

This was a mild case—treated in its incipiency with nitric acid most thoroughly. The ulceration had not extended so deeply, nor so far beneath the margin of the skin, as to make it almost impossible to reach every portion of the diseased surface.

There was no scorbutus. The gums were firm and hard. The patient was very pallid, his heart feeble, and his pupils dilated. The mucous membranes were very pale, and the expression of the face haggard and anxious.

He recovered rapidly, with a good stump, and was transferred to St. Elizabeth Hospital, May 4th, to enable him to procure an artificial limb.

This man was seen several months after walking with great ease on his artificial leg.

Case II. L. D. Thurston, private, Co. A, 16th Regiment New Hampshire Vols., aged 42, was struck by a fragment of shell on Dec. 13th, 1862, at Fredericksburg, Va., which caused a severe but superficial wound of the integuments on the outer side of the left thigh.

When seen, Feb. 17th, 1863, there was a wound at the middle of the thigh, on its outer aspect, three and a half inches long by two and a half wide, exposing the muscular tissue slightly, the surface of which was glazed and dry.

On the 10th of February, it had been found desirable to open an abscess, three inches below the left greater trochanter. On the 13th, this had assumed an unhealthy look, and when I saw it on the 17th, the incision made by the lancet, half an inch in length, was surrounded by a border of sphacelus one inch in width, and by an areola of purple congestion, in which there seemed to have occurred a complete stasis of the circulation. There was no pus, but a discharge of very fetid, dark-colored serum. There was no swelling, ulceration, or eversion of edges of the incision, which, although mortified, remained as sharp as when first made.

There was profound nervous prostration, which was indicated by his rapid, feeble, and irritable pulse; by his sallow hue; his haggard and anxious expression of countenance; his weary and helpless decubitus, and great mental despondency.

He was treated internally with stimulants, the most condensed and nourishing food, and citrate of iron and quinine; nitric acid was applied locally, followed by a weak solution of creasote, three drops to the ounce of water, as an antiseptic dressing.

The sphacelus extended in all directions rapidly, unchecked by this treatment, from which I hoped little, since it was impossible to bring the acid into contact with the diseased tissues, although it was injected into the incision. The constitutional symptoms, also, became more grave.

On the night of the 20th, there was quite a severe hemorrhage from the incision, oozing slowly and very difficult to restrain, since it was caused by the erosion of vessels at a distance from the small incision. There was now a circular patch of sphacelus surrounding this small incision, three inches in diameter.

On February 23d, he was removed to the ward in the brick building. The original wound, hitherto unaffected, now began to be black and offensive. The sphacelus extended from these two centres, at the rate of one inch daily, preceded by the above-mentioned areola of purple statis.

No treatment, local or constitutional, produced the least effect. Stimulants were given in every possible form, until the stomach refused to retain them. Pure nitric acid was freely applied to the diseased surfaces with no benefit. He fell into a typhoid condition, with muttering delirium, subsultus tendinum, etc., and finally expired February 28th.

The sphacelus then extended from the trochanter major to three inches above the outer condyle; and from the median line in front to a corresponding point behind. There had never been any ulceration, but the tissues seemed to perish *en masse.* The incision made by the lancet was yet plainly seen in the centre of an extensive surface of mortification. This man was 42 years old, had had chronic diarrhœa, and was in a feeble state of health when wounded.

No benefit was observed from any treatment. He took, in addition to the nutrients and tonics, the acid mixture with tincture of opium.

The preparation was forwarded to the Army Medical Museum.

Case III. Private Samuel Fossett, Co. I, 12th Regiment Rhode Island Vols., suffered a compound fracture of the right clavicle, by a fragment of shell, at Fredericksburgh, Va., December 12th, 1862. There was very extensive injury to the soft parts. He was admitted to this hospital December 26th.

On February 18th, 1863, a large granulating surface, two inches deep, was covered with an ashy, gray slough, and the discharge was converted from pus into an offensive ichor; the edges were thickened and everted, and surrounded by the characteristic areola of congestion, fading away into a bronzed hue.

The ulceration here attacked cicitricial tissue, which disappeared very rapidly. The entire surface was treated with nitric acid diluted, and tonics and stimulants were given internally.

The acid changed at once the character of the sore. It soon commenced to granulate, and the repair was then as rapid as had been the destruction. He was discharged the service, April 7th, for partial paralysis of the right arm, from traumatic injury of the brachial plexus.

This case was easily treated, being recognized as gangrene at once, and terminated quickly in convalescence, with a perfect recovery. The constitutional symptoms were never severe, although the man was anæmic, and had great mental despondency.

He was also removed from the crowded ward, and placed in a more salubrious one in the brick building.

CASE IV. Charles Underwood, Co. D, 31st Georgia, aged 24, for a wound caused by a shell, on December 13th, 1862, at Fredericksburgh, Va., suffered an amputation of the left leg at its middle.

He entered this hospital December 25th. Nothing interesting occurred until February 20th, 1863, when a granulating surface on the face of the stump became covered with a gray slough, and the discharge was changed from pus to an offensive ichor. He was anæmic and much depressed in spirits. This case occurred in the same ward, but on the opposite side from the previous ones. The ulcer was dressed with sol. of creasote, and tonics and stimulants given internally.

On the 23d he was removed to the brick building, his ulcer freely treated with nitric acid, and then dressed with a mixture of equal parts of bals. copaibæ and ol. ricini. The ulceration had never been rapid in this case, and it was only considered specific because other undoubted cases had occurred in the same ward.

The character of the ulcer soon became changed, the granulation was rapid, and he was finally transferred to military headquarters, May 4th, 1863, perfectly recovered.

CASE V. Isadore Wick, Co. D, 1st N. Y. Artillery, aged 32, had his right thigh amputated on the field, for a comminuted fracture of the tibia, received December 13th, 1862, at Fredericksburgh, Va., caused by a minie ball.

He was admitted December 26th. I regret that I can give no account of his progress, but I learned that the flaps had been insufficient, and that the stump had been closing by tedious granulation. There had been a free discharge, and his general condition was, therefore, unfitted to withstand the depressing influence of hospital gangrene. His bed was on the same side of the ward, and in close proximity to the first case reported.

The operation had been a circular one; the grannlation had entirely covered the end of the bone, and there was, when seen on the 18th February, only a narrow strip, not yet cicatrized, between the margins of the skin. This was now covered with a gray slough, and had the characteristic fetid odor. It was dressed simply with the creasote solution, my faith in the acid treatment having been shaken by the reports of medical officers who had visited cases at Annapolis, Md.

*Feb. 23d.* The case was now considered as an unequivocal one of hospital gangrene, and was removed to the ward selected for such cases. The cicatricial tissue had all yielded to the sloughing, and the subcutaneous connective tissue had been destroyed for two inches beneath the skin at the outer angle of the original incisions. No change was made in the local treatment, as the creasote was a perfect deodorant, and as good results were hoped for, from its local use, as had been reported from Annapolis.

The destruction was limited to the connective tissue until March 9th, when there was a margin of sphacelus half an inch wide in the true skin. The constitutional symptoms had been growing more grave. His mental despondency was most marked, his face pale and anxious, his pupils dilated, his pulse 100 per minute, and feeble, and his skin very moist; a free diarrhœa had also commenced.

The tonics and stimulants having produced anorexia and nausea, were replaced by a mixture of tincture of opium and hydrochloric acid, in such proportions that he took gtt. xvj of tr. opii, and gtt. iv of acid. hydrochlor. every fourth hour. Beef essence and milk punch were given as freely as his stomach would tolerate them.

*11th.* As his general condition became less favorable, the local action was changed from molecular death to sphacelus. The whole face of the stump has now a margin of black mortification of the skin, outside of which was the usual areola of purple congestion—the complete stasis of to-day becoming the sphacelus of to-morrow. The end of the femur, protected by rosy granulations, now protrudes from the black mass of sphacelus, the integument having become loosened by the destruction of the subcutaneous connective tissue and retracted. The presence of this mass of putrefaction seems to add to the nervous prostration—if indeed the absorption of such peccant material is not its sole cause. Pure nitric acid had been applied several times, but it had been found impossible to convey it into the depths of the ulcer.

*21st.* No change except for the worse had occurred. Stimulating poultices of cinchona, ginger, and flaxseed had been used locally, but with no benefit.

Antiseptics, such as the solutions of the chloride of soda, creasote, and permanganate of potash were necessary to purify the ward and render it endurable for his attendants. Nutrients and stimulents had been pushed to the last extent, and opium had been largely given for its supposed specific effect in the disease, as well as to allay suffering. The symptoms had been typhoid for several days; emaciation had gone on rapidly; there had been subsultus tendinum and muttering delirium with extreme prostration until this date, when death occurred.

The limb was removed after death and the specimen sent to the Army Medical Museum. The sphacelus had involved all the tissues for five inches above the divided bone, and there seemed to have been a faint effort to form a line of demarcation.

This was, at first, a very mild case, with no very decided constitutional depression, until the system seemed to be poisoned by the absorption of the products of the gangrene, when the ulceration became more rapid, and was finally, as the strength succumbed, converted into uncontrollable and rapidly extending sphacelus, accounting satisfactorily for the unfortunate result.

The treatment locally had been, first, weak sol. of creasote, gtt. iv to water ℥j, made soluble by alcohol ʒj; and second, strong nitric acid; never fully applied, however, to the depths of the deceased tissues. The constitutional treatment was stimulating, sustaining, and tonic.

Case VI. Patrick Morrisey, private Co. K, 20th Mass. Vols., aged 46, had been wounded on the 13th Dec., at Fredericksburg, and suffered an amputation of the left leg, at its middle.

He was admitted Dec. 26, 1862. There had been no union; the flaps had sloughed, and the bone protruded, as in almost every case transported hither on that occasion. He had been broken down by a free discharge and diarrhœa. His bed was in the same ward, and on the same side with the previous cases, and his wound was dressed by the same medical officer and nurses.

*March 6th.* His leg, for six inches above the face of stump, was swollen, erysipelatous looking, and riddled with small orifices, leading to abscesses. The face of the stump was composed of purplish, lowly organized, cicatricial tissue, upon which a patch of sphacelus, as large as a nickel penny, surrounded by the livid purple areola, now appeared. This was freely incised, touched thoroughly with pure nitric acid, and dressed with a fermenting poultice sprinkled with sol. sodæ chlorinant. Internally, gtt. xv of McMunn's elixir of opium were given every fourth hour.

This attack was ushered in by nausea, rigors, and fever; and the patient had a desponding, yet anxious expression of countenance. His diet was "extra" with stimulants. He was at once removed from the large ward and placed in a pleasant one selected for these cases, on the second story of the brick house.

*9th.* The edges have yielded until the ulcer is now two and a half inches in diameter, covered with a tough yellow slough, which adheres strongly and extends deeply into the tissues of the centre. The pure acid was again thoroughly applied, both to the diseased surface and to the areola. Internally, a mixture of tr. opii, gtt. xvj, acid. hydrochlor. gtt. iv, every three hours, with extra diet and stimulants, was ordered.

*12th.* Ulceration was unchecked, was now three by two inches in extent, and the margins, undermined by destruction of connective tissue, were "piled up," thickened, and everted. There was diarrhœa, with decided nervous prostration. The sloughs were all removed with the forceps and scissors, until the vascular tissue beneath was reached, the sore, well dried, was thoroughly cauterized with nitric acid, and dressed with a poultice and disinfectants. Elixir vitriol was ordered for the diarrhœa.

*13th.* The appearance of the sore was improved, and the general condition was better.

*14th.* Granulations were now seen on the sore, and the areola had lost its livid hue.

*April 20th.* An immediate improvement in the general condition followed the thorough acid treatment of the ulcer. The tongue became clean, the diarrhœa was checked, the appetite returned, the food was assimilated, the whole expression of the countenance was changed from anxiety to cheerfulness, the gnawing pain was relieved, and the patient slept soundly. The appearance of the sore was also changed as suddenly from a foul, offensive, sloughing ulcer, to a rosy, granulating surface, in which repair was as rapid as the previous destruction.

This man recovered with a good stump, and was transferred to St. Elizabeth Hospital, to receive an artificial leg.

CASE VII. John Jordan, private, Co. H, 2d Maine, aged 20, was struck at Fredericksburg, Va., Dec. 13th, 1862, by a fragment of shell, which passed across the right thigh below Poupart's ligament, through the scrotum, destroying the right testicle, and behind the left thigh, producing in its course very extensive but superficial wounds of the anterior portion of right, and posterior portion of left thighs. He was admitted Dec. 26, 1862, and placed in Ward No. 5.

*Feb.* 27, 1863. He had febrile disturbance and anorexia, a yellow furred tongue, an anxious, restless expression of countenance, and a burning pain in his left thigh. There was, on the right thigh, a granulating surface three by two inches in dimensions, level with the integument and cicatrizing rapidly; a smaller, equally healthy surface remained unhealed on the scrotum; whilst on the posterior portion of the upper part of the left thigh, an ulcer, three by two inches in extent, was found, oval in shape, covered with an ashy, gray slough, with its margin thickened and everted, surrounded by a livid areola, and, instead of normal pus, discharging a thin fetid serum mixed with debris. He was at once removed to the house, the whole diseased surface was touched with pure nitric acid, and dressed with creasote lotion: stimulants and the best extra diet, with beef essence and milk punch at short intervals, and ferri. et quin. citrat. three times daily, were ordered.

*March* 5. The attempt to push the nutriments and stimulants produced, as it generally does, anorexia, nausea, vomiting, and diarrhœa. The tongue became thickly coated with a yellow fur, and dry and red at the tip; and so great was the gastric disturbance, that all medicines were discontinued, and the stomach allowed to recover its tone by rest, no longer being teased either by drugs or excessive and undesired nutriment.

No benefit followed the local application, and the ulceration had extended in every direction. There was the characteristic margin, preceded by the areola of livid stasis, preparing the tissues for their rapid destruction. The connective tissue beneath the skin had been destroyed, so that the skin for one inch from its margin was perfectly movable. The muscles separated from each other by the death of their connective tissue lay in the wound, bathed in its discharge, but rosy and florid, and resisting the advance of the disease.

This sore was so unmistakably hospital gangrene that several pictures of it were taken by direction of Surgeon Brinton, which represent well the surface of the ulcer, dripping with its thin serous discharge mingled with shreds of dead connective tissue, its "piled-up," thickened, and everted margin, surmounted by a thin line of vivid redness, and its broad zone of purple congestion, shading away into a bronze hue; the depth of color in the areola indicating the engorgement of the small vessels, and its hue, the feebleness and slowness of the movement of the blood.

It was determined to try the opium treatment, with hydrochloric acid as a tonic, and this mixture was given in the proportion of tinctura opii gtt. xvj, with acid. hydrochloric. gtt. iv, every three hours. The sore was dressed with a stimulating poultice composed of flaxseed, cinchona, and ginger mixed with porter.

11*th.* Under the use of the acid internally, the tongue had become clean and moist, the tone of the digestive apparatus improved, and a fair quantity of food had been taken. Porter and ale had been given as the stomach would retain them. But little change had taken place in the character of the ulcer, which was eight inches in length by seven in breadth, extending to the perineum, and irregularly oval in shape. The muscles exposed (the semi-membranosus and biceps) had yielded, and were now almost divided. The sores on the right thigh and scrotum had not been in the least affected, *but were cicatrizing rapidly.*

The entire surface of the gangrenous sore was now thoroughly cleaned, all sloughs and shreds removed with forceps and scissors, was well dried with lint and carefully painted with pure nitric acid. The brush, charged with acid, was passed beneath the excavated margin, in some places more than an inch. The patient was etherized, and this acid application was made most carefully and completely. This was considered a *dernier resort*, for, although the capacity for taking and assimilating food seemed to have been increased by the acid treatment internally, yet his strength was daily diminishing from the exhausting discharge and from the absorption of the products of the gangrene.

On the succeeding day an entire change in the sore was observed; there had been no extension of the gangrene, the fetid odor was gone, and the discharge was more consistent and less serous. In a few days more all the shreds of dead fascia were removed, and the surface was found to be perfectly healthy. The contrast between the ragged, offensive, yellow-colored ulcer, before the last application of acid, and the florid, perfectly normal, granulating surface, which replaced it, was as gratifying as it was surprising. With the local there was also a constitutional improvement. The appetite became voracious; the patient slept well; there was no pain, and the process of repair was very rapid. The acid was continued internally.

*April* 1. The sore was now two by three inches in extent, and cicatrizing rapidly.

20*th.* But a small surface yet remained unhealed. The patient was in perfect health, had gained flesh very rapidly, and was now on crutches.

There was some contraction of the flexors, as the biceps and semi-membranosus were both involved in the destruction.

The most remarkable circumstance in this typical case is the fact that when the gangrene attacked the granulating surface of the left thigh, the equally large granulating surface of the right thigh was unaffected; and that whilst the gangrene was ravaging the left thigh the rapid cicatrization of the right proceeded uninterruptedly. The discharge from the left thigh was so profuse that no precaution would have prevented the virus from coming in contact with the excoriated surfaces of the scrotum and right thigh. If, therefore, the disease be propagated by inoculation, all the circumstances were favorable; since the proximity of the thighs at their upper part, and a denuded surface on the scrotum, that might act as a link, render it certain that a portion of the great discharge from the left must frequently have been placed in contact with both of the other sores. If, on the other hand, the gangrene be not a local but a constitutional disease, why should it spend itself on one granulating surface when there were two others equally obnoxious? The contrast between these sores was marked; for whilst the tissues of the left thigh were melting away under one's very gaze, the process of repair in the scrotum and right thigh was progressing as rapidly as under the most favorable circumstances. In its earlier stages this case was twice treated with nitric acid, and perhaps imperfectly, from its not having been carried into the recesses of the ulcer.

The system of urging nutrients, stimulants, and tonics, irrespective of the natural desires of the patient, is, I am satisfied, pernicious. The vital energy being depressed, the digestive organs are enfeebled; and the introduction of milk-punch, beef-essence, egg-nog, &c., with stimulants, porter, ale, &c., into an unwilling stomach, simply produces anorexia, nausea, vomiting, and diarrhœa. The tongue became furred and dry, and there was a perfect disgust for all food. The hydrochloric acid was given to correct this condition; and whether its action was confined to the stomach in rendering soluble the aliment, given in very small quantities, or whether its action was catalytic or eliminative, I shall not pretend to state, but under its influence the tongue became moist and clean, and the patient made known his desire for food. Nutrients were then given more freely as his appetite returned. The recovery of this man is due, first, to the thorough application of the nitric acid to every portion of the ulcer, which changed its specific nature immediately; and, second, to the combined effect of acid and opium internally. After the last complete application of acid the sore was changed in character, the areola disappeared, the surface became clean, the margins lost their elevated appearance, the serous discharge became purulent, and the offensive odor was entirely destroyed. An equally marked improvement occurred in his general condition.

CASE VIII. R. W. Plummer, private, Co. D, 1st Delaware Vols., aged 34, was struck on the outer aspect of the left thigh, at its middle, by a bullet, at Fredericksburgh, Va., Dec. 13th, 1862. The bullet struck against his bayonet, and penetrating his leg caused a severe flesh wound.

He was admitted Dec. 26th, 1862. The ball dropped out Jan. 1st, 1863. This was the only case of gangrene that occurred in the brick building, and appeared on the upper floor, at a distance and entirely disconnected from the wooden pavilion where the other cases were treated.

*Feb.* 15. The wound was almost closed.

*March* 9. For three days past there have been anorexia and fever. There is now an ulcer on the left thigh as large as a silver quarter dollar, with everted edges, livid areola, and a fetid discharge. The connective tissue has been removed from beneath the margins by ulceration, leaving the skin undermined. Pure nitric acid was applied to the ulcer, which was then dressed with solution of sodæ chlorinat., and tinctura opii and acid. hydrochloric. given every three hours internally.

12*th*. The ulceration having continued unchecked, nitric acid was again applied, followed by a poultice; the man was moved into the gangrene ward.

15*th*. The second use of the acid locally has changed the whole character of the ulcer. It is now about two inches in diameter, free from odor, rosy in color, and granulating rapidly.

22*d*. This improvement continued until this date, when the repair had brought the bottom of the ulcer to a level with the surrounding skin, and cicatrization had fairly commenced. There was at this time a profuse diarrhœa from some error in diet, with depression of the vital energies, which was accompanied by a relapse into the gangrene. The sore is now covered with a gray slough, is very painful, and has the characteristic areola. The expression of the patient's face is very anxious and haggard. Nitric acid was most thoroughly carried into the recesses of the ulcer, and the acid and opium mixture ordered internally.

28*th*. The ulceration has extended very rapidly, and is now a perfectly characteristic specimen of hospital gangrene. The local use of the acid seemed to do harm, since the destruction of tissue was more rapid after its application. The general condition is alarming. There are uncontrollable diarrhœa and nausea. The pulse is very rapid and feeble, the skin relaxed, the mind dejected; and the expression of face, and the decubitus betoken profound debility. Nitric acid was again most carefully applied.

*April 2d.* The ulcer now measures seven by six inches. The whole surface is dripping with a fetid, sanious discharge, laden with debris of the fascia and connective tissue; the skin, undermined, is separated from its attachments for the distance of one and a half inches from the free margin, which is "piled up," everted, and surrounded by an areola of livid, bluish redness, three inches in width. The general condition is most unfavorable. There are great prostration, profuse perspiration, and diarrhœa; with a pulse of 130 and very feeble, and tongue red and dry; and a complete disgust for food, with nausea. His condition is so desperate, that I feel no hope of his recovery. The nitric acid, when last used, had only hastened the destruction of tissue, and after its failure, I was without a substitute. It had been twice used with no good effect, and had been applied most carefully to the entire surface. The nervous prostration, which is so prominent a symptom of this disease, was so profound that I think this man would have died within 48 hours, exhausted, but for the treatment employed. The constant burning pain had destroyed his rest; his diarrhœa was profuse, his skin bathed with perspiration, his stomach so irritable as to reject all nutrients and stimulants, and the expression of his face was dejected, depressed, anxious, and haggard to a remarkable extent.

The surface was again carefully cleaned of all sloughs and discharge, and thoroughly cauterized with the solution of bromine, employed by Surgeon Goldsmith, U. S. Vols., viz.:—

Bromine, ℥j; potassii bromid., ʒiij; aquæ, ℥iij. The patient was anæsthetized, and the caustic solution applied most liberally to the entire diseased surface, which was immediately deodorized, and covered with a tough yellow deposit. The sore was then covered with a piece of dry lint, a second piece of lint, wet with the bromine solution, was placed over it, upon this a piece of muslin spread with cerate, and over all a piece of oiled silk, confined closely with a roller, was bound; and the local effect of bromine was thus continued in the form of vapor. The burning pain disappeared; the antiseptic effect of bromine seemed to restore the appetite by destroying the virus that had been poisoning the whole system, and, for the first time for many days, he took some food.

*May 4th.* The exposure of the surface of the body to the air at this application of the bromine was followed by a severe attack of bronchitis, with vicid and bloody expectoration, and severe dyspnœa, which was treated by warm cataplasms to the chest, and by expectorants.

*7th.* The ulceration has ceased, the odor disappeared, and the surface is now clean and rosy. The vapor has been used twice daily. An immediate improvement followed the use of bromine, both locally and constitutionally. The pain, diarrhœa, and profuse perspiration ceased, the appetite returned, and the nervous prostration soon yielded to the extra diet and stimulants. The bromine was used twice in solution, and continued in the form of vapor until the granulations became normal.

*23d.* He is convalescent, and the ulcer entirely cicatrized. The bromine produced this gratifying change, after the entire failure of nitric acid; nor do I hesitate to affirm that, but for its employment, the case would have terminated fatally.

Case IX. George Zilch, Sergeant, Co. K, 7th N. Y. Vols., aged 25, had his left leg amputated at its upper-third, for a bullet wound received at Fredericksburgh, Va., Dec. 13th, 1862.

He was admitted to Douglas Hospital December 26th, and placed in Ward No. 5. The stump had closed slowly by granulation, until there remained an ulcer as large as a half dime on its face.

*April* 14, 1863. This ulcer was inflamed around its edges, and covered with a white pultaceous slough; there was no constitutional disturbance, and the patient was allowed to remain on his crutches. The ulcer was cauterized with nitric acid, and dressed with solution sodæ chlorinatæ.

*18th.* The ulceration and areola are both enlarged, and the slough, yet very tough, is thicker. Acid was again used locally.

*21st.* He is feverish, and inclined to nausea; his pulse 120, skin hot, and tongue thickly coated. The ulcer is extending in depth, and he was removed to the gangrene ward.

*22d.* The solution of bromine was applied to the sore after cleansing the surface as much as possible of the tenacious slough. His general condition was unfavorable, and there was a tendency to diarrhœa and perspiration. Internally he took muriatic acid, with extra diet and stimulants.

*24th.* Bromine was again applied to the surface, and used in the form of vapor. The ulcer was now three inches in diameter, irregularly circular in form, with ragged, everted, and thickened edges, and surrounded by a purple areola. The slough was one inch in thickness, and resisted the action of the bromine.

*26th.* The sore is looking better; it has been disinfected since the first application of the bromine, and the constitutional symptoms are better.

*27th.* A painting in oil was made to-day, by Surgeon Brinton's direction, which would be pronounced a good representation of hospital gangrene.

*28th.* The slough is much thinner to-day, and the granulations are showing through the thin gray covering.

*29th.* The sore is much better, is becoming covered with granulations, and has lost almost entirely its specific appearance. There is no constitutional disturbance, no fever, no headache; the tongue is cleaning off, and there is a return of the appetite. The bromine vapor was discontinued, and a sol. sod. chlorinat. substituted.

*30th.* The livid areola has been changing daily in hue under the bromine treatment, has now entirely disappeared, and the sore is perfectly healthy.

*May 6th.* He is still improving, and is taking tonics and nutrients.

*20th.* The sore is reduced to half its original size, and is now cicatrizing rapidly.

*24th.* There is now a surface as large as a penny unhealed. The patient's health is very good; he is about the ward on crutches, and is no longer considered an interesting case.

We have in this case another instance in which the acid, locally used, proved useless, and in which the solution of bromine caused an immediate improvement. It was found necessary to apply the caustic solution to the ulcer three times, owing to the thickness of the slough, which was too closely attached to be removed by spatula or forceps. Its action here seemed to be to correct the fetor at once, to check the molecular death, and to change the hue of the areola, by causing a more healthy action in the capillaries. The gnawing, burning pain was relieved, and the patient was able to sleep in comfort. The absorption of the virus, produced in the ulcer was prevented by its destruction, and the nervous system quickly regained its tone.

This man steadily improved, recovered with a good stump, and was finally sent to New York to be mustered out of the service.

I will conclude this brief clinical history by a short summary.

*Etiology.*—This disease made its appearance in a wooden pavilion, containing fifty beds, most of them occupied by very seriously wounded men unable to leave the building, with a cubic capacity of 1050 feet to each bed, heated by ordinary radiating coal stoves, devoid of any system of ventilation, and having no ingress for pure air, nor egress for foul, except through the windows and doors. This want of pure air was combined with a want of strict police, and a careless and unscientific method of dressing the wounds, rancid ointments being largely used instead of the ordinary water dressing.

No case of gangrene was received as such into the hospital, nor is it probable that it was otherwise introduced.

Although a majority of the cases in this ward escaped gangrene, yet there was evidently some depressing agent at work, since but few wounds healed rapidly. The patients seemed also dispirited, homesick, and moody. Those who were attacked were removed to a ward in the brick house, where they were isolated, and at the same time placed under better hygienic influences.

Two shafts for foul air, connected with the stoves, which withdrew the foul air from near the floor, had recently been placed in the ward by order of the Surgeon-General. This was not considered sufficient, and the long doors of these foul air shafts were kept constantly open. Strict attention to cleanliness and careful dressing was enforced, and, what might have been a very severe epidemic, was confined to few cases. The upper row of windows were rehung in such a manner as to direct the currents of cold air admitted in a line with the roof, and, to crown all, the *ridge ventilation* was applied to the pavilion.

These precautions, and an improvement in the diet of the house, giving more vegetables and antiscorbutics, enabled me to prevent any further serious manifestations.

| No. | Names. | Ward | Side. | Date of attack. | Grade disease. | Resu | Treatment. |
|---|---|---|---|---|---|---|---|
| 1 | Otto Kossacke | 5 | West | Feb. 15, 1863. | Ulceration | Cured | Nitric acid. |
| 2 | L. D. Thurston | 5 | " | " 15, " | Sphacelus | Death | " |
| 3 | Sam'l Fossett | 5 | " | " 18, " | Ulceration | Cured | " |
| 4 | C. Underwood | 3 | East | " 20. " | " mild | " | " |
| 5 | J. Wick | 5 | West | " 28, " | Sphacelus | Death | " |
| 6 | Pat. Morrissey | 5 | " | March 6, " | Ulceration | Cured | " |
| 7 | J. Jordan | 5 | East | Feb. 27, " | " | " | " |
| 8 | R. W. Plummer | 1 | Building | March 9, " | " | " | Bromine. |
| 9 | Geo. Zilch | 5 | East | April 14, " | " | " | " |

There were three other wards of like construction, with Ward No. 5, but two of them were unoccupied, and the third one contained fewer, and less serious cases.

The foregoing table indicates that of the nine unmistakable cases reported, eight appeared in Ward No. 5; and that of these eight five occurred on the west, and three on the east side of the ward. Although these facts would suggest the probability of inoculation, yet I cannot but remember that there were at least thirty-five other wounded men in this ward who escaped, although the disease had been in existence several days before Feb. 17th, when I took charge, and no precautions against contagion had been employed.

I shall not attempt to argue here the long-disputed point of contagion or *non*-contagion, but simply state it as the result of my observation, that I saw no well-marked instance of inoculation, whilst I did see many wounds escape this influence where inoculation was not only possible, but probable. Nor was it necessary to invoke the aid of any specific virus, since the unfavorable hygienic influences which had surrounded these men from the date of injury, Dec. 13th, and the date of admission into hospital, Dec. 26th, 1862, to Feb. 15th, 1863, were sufficient to produce, in all the cases treated in Ward 5, a well-marked cachexia—neither scorbutus nor anæmia, but more unmanageable than either, and due, most probably, to the absence, simply, of fresh, pure air in sufficient quantities. With no further improvements than those mentioned, a marked change for the better took place in the other inmates of this ward. Their wounds became healthy, and healed rapidly, and their spirits became cheerful.

It will be observed, from the table that I have described, two grades of hospital gangrene, the one mild, generally manageable, and characterized by ulceration or molecular death of the tissues, spending itself generally in the subcutaneous and intermuscular connective tissue; the other, more rapid in its course, more fatal, less amenable to treatment, and distinguished by sphacelus or mortification, *en masse*, of the invaded tissues. It will be seen that of the nine cases, seven are described as ulceration, and two sphacelus, and the latter both fatal.

These two cases last mentioned were entirely uninfluenced by the treatment employed. The rapid invasion and advance of the mortification, and the impossibility of reaching it with nitric acid, to influence it locally, left but little to do, but to observe its profoundly depressing effect on the vital forces. Bromine may prove the antidote in such cases, but its virtues were then unknown to me.

*Treatment.*—Recognizing the depressed condition of the first few cases, I endeavored to remedy it by giving at short intervals nutrients and stimulants, with such tonics as seemed proper; and milk-punch, alternating with beef-essence, porter or ale, and egg-nog, was at first given, regardless of the desires of the patient. The ferri et quin. cit. with sherry wine was given in doses of gr. vj to x three times daily. This system was found injudicious, since it overpowered the feeble digestive organs, and caused nausea, vomiting, and diarrhœa; it was suspended, and acid. hydrochloric. gtt. iv, in combination with tr. opii gtt. xvj, were given every three hours. Under this treatment the tongue became moist and clean, and the appetite returned sufficiently to cause the patient to ask for and enjoy a reasonable quantity of food. The opium was given to allay the gnawing pain, and to give rest and sleep, as well as to obtain any specific influence over the disease which it might possess, as claimed for it by the older writers.

The local treatment consisted at first in the use of undiluted nitric acid, freely applied to the entire surface of the ulcer. The table indicates the success of that treatment. Of the nine cases, all were treated with the acid in the early stages. Of these, two were fatal, and two resisted the acid; or, in other words, in four cases it was useless. Of these four, the two fatal cases were not treated otherwise, whilst the other two were treated with success, with bromine. The five cases treated successfully with acid are marked ulceration, and were milder than those that proved fatal, or than those that were treated with bromine successfully.

The dressing, after the use of the acid, was an antiseptic wash, either of creasote or liq. sod. chlor. in a dilute solution. In some instances a yeast poultice, or a stimulating one of cinchona, ginger, and porter, seemed to assist in cleaning the surface.

Where the sore lost its sloughing character, after the use of nitric acid, the mild antiseptic washes were sufficient to encourage rapid granulation.

Of more value than the acid is the solution of bromine in water and bromide of potassium, proposed by Surgeon Goldsmith, U. S. Vols. Two cases were treated with this agent, in both of which nitric acid had failed. One of these would, doubtless, have proved fatal, whilst the other was progressing rapidly, although the acid had been used several times without benefit.

The action of the bromine is that of a caustic; all the necrosed tissues are converted into tough yellow shreds, and are perfectly deodorized. The ulceration seems to be checked at once, while the nervous system, no longer depressed by the absorption of the fetid products of the mortification, soon recovers from its depression. The areola loses its livid hue, becomes more crimson, and finally disappears; the sloughs are rapidly thrown off, and a rosy, florid surface appears bsneath.

The bromine was also used in the form of vapor, confined to the surface by oiled silk. Its antiseptic influence is very powerful, since not the least odor could be perceived on dressing these gangrenous sores, even when they had been covered closely with oiled silk for twelve hours. From its antidotal efficacy in these two cases, I have formed a high opinion of its value in the local treatment of this disease.

*Microscopy.*—The discharges from several of these cases were examined, to ascertain whether some of the speculative views in regard to the presence of fungi and their influence in producing the disease could be sustained, but no fungi were found.

The discharge consisted of fluid, granular matter, and debris. The connective tissue seemed to have been broken down into unrecognizable granular material. The fibrous tissue was softened and easily teazed out, and in the muscular tissue the striated appearance was lost before the fibrous.

No evidence of textural growth was found in the discharges, although the "piled-up" and thickened margins of the ulcers would probably reveal, on examination, a multiplication of the connective tissue-corpuscles, as reported in a similar group of cases at Annapolis, Md., by Assistant Surgeon Woodward, U.S.A.

Since the preceding report was drawn up, four other cases of hospital gangrene have been observed, occurring sporadically, and treated with success with bromine. Their clinical histories are very briefly offered for consideration, in addition to those already submitted. No other cases have occurred in this hospital. It will be observed that three of these four cases were fully treated with pure nitric acid without benefit, and that the four did yield eventually to the local application of bromine. Where that remedy has required heretofore several repetitions, it would now be used more energetically. The diseased surface would be thoroughly cleansed of all sloughs, by removing those portions dead, yet tenaciously adherent, with the forceps and scissors, and pure bromine would be freely used by means of a glass pipette or a syringe. A number of the cases reported I am now satisfied would have proved fatal but for this local treatment; and it will be a proud satisfaction to Surgeon Goldsmith to know that he has not only already been instrumental in preserving so many valuable lives, but that he has provided the military surgeon with a defence against one of the most deadly and obdurate of his antagonists.

In conclusion, the writer regrets that circumstances do not permit him to render these clinical histories more acceptable to the critical reader. The report was made to the Surgeon General for the future use of the surgical historian of the war, and now meets the public eye in this crude form because it has been represented that further evidence as to the virtues of bromine might be of service in the exigencies of the coming campaign.

CASE X. Michael Flood, age 24, priv. Co. D, 33d N. Y. Vols., was struck by a minie ball at the battle of Chancellorsville, May 3d, 1863, which entered the right thigh anteriorly, comminuted the femur for four inches at the junction of the middle and upper-third, and escaped posteriorly. He was admitted on the 8th of May. This man had been suffering from diarrhœa, and was in a feeble condition. The usual profuse suppuration followed the injury, thus reducing his strength still further. The leg was lightly dressed, was supported by sand-bags, and extended by means of a brick attached to the limb by a band of adhesive plaster. On July 23d, the inner surface of the thigh, at its middle, was found red and inflamed opposite the seat of fracture, and in the centre of this diffused redness there was a spot very tender on pressure, slightly softened, and indicating an approaching abscess. This was treated with a poultice. On the 24th, a small black spot of sphacelus was observed in the centre of this inflamed region, which was easily detached, and then revealed a sloughing condition of the connective tissue.

No previous abrasion of the skin existed, nor any wound which would have permitted of inoculation; nor was there any other case of gangrene then under treatment. The diarrhœa simultaneously increased, and the patient's condition became rapidly worse. The ulcer was treated with the solution of bromine and bromide of potassium. This was followed on the following day by pure nitric acid, with the apparent effect of adding to the rapid destruction of tissue. On the 27th, the ulcer was in dimensions five by four inches, with irregular, ragged edges, everted, thickened, and extensively undermined at the margins; and, in a word, presented the characteristics of severe hospital gangrene. It was now thoroughly treated with pure bromine. The improvement was marked; the ulcer became deodorised; lost rapidly its sloughing, ragged look; became clean, then florid, from rapid granulation, and in a few days was converted into a perfectly healthy, rosy surface. The man's broken condition gave no hope of his recovery. There was still a free discharge from his fractured femur, his diarrhœa continued unchecked, and from these he sank exhausted, and died on the 6th of August. The gangrene here did not attack the gunshot wound through which the discharge from the seat of fracture escaped, but invaded a new unwounded surface, and one upon which no abrasion was known to exist. The gangrene was perfectly removed previous to his death.

CASE XI. P. C. Barton, Co. D, 26th New York, age 25, was struck at Chancellorsville, May 23d, 1863, by a piece of shell, which comminuted with extensive laceration the right tibia and fibula, at the middle, and fractured the right femur at the junction of the lower and middle-third. This was a most severe wound, since the soft parts were so greatly injured. He was admitted on the 8th, his leg placed in a bran-box, and slight extension attempted by fastening the foot to the foot-board and elevating the lower end of the bedstead. Profuse discharge and occasional attacks of diarrhœa reduced his strength, whilst his wounds were undergoing rapid repair. On July 1st, he was removed to a ward filled with other wounded men, and in a few days the granulating wound on his leg became grayish, and the discharge thin and offensive. The wound of the thigh had almost closed. This was recognized as mild hospital gangrene, and was treated with strong nitric acid, followed by yeast poultices. No improvement followed. The cicatrized tissue, the offspring of the previous repair, was rapidly removed, the ulceration spread under the sound integument, and the sore assumed the characteristic appearance of gangrene. Stimulants and mineral acids were given internally, but rapid emaciation and general prostration ensued from the continuance of the diarrhœa. The sore was then treated with the solution of bromine, but required several applications to check the ulceration. Pure bromine would now be employed in such a case. This man was moved to a better ward on July 30th, and the ulcer was then seven inches in length by four in width, occupying the outer side of the right leg.

Soon after his removal an abscess formed in the outer portion of the right thigh, the pus was evacuated, and gangrene immediately attacked the walls of the cavity; this extended with great rapidity, became four by three in extent in two days, and was immediately arrested by the bromine. No hope whatever was entertained of this man's recovery. A severe comminuted fracture of both bones of the leg, a compound fracture of the same femur, and two severe attacks of gangrene, combined with an obstinate diarrhœa, had brought him to the last stages of exhaustion. By the 12th of August his ulcers were normal in appearance, and were granulating rapidly; the bones had united, and his diarrhœa had been checked by nitrate of silver and opium. The improvement was gradual, but constant; his stomach recovered its tone, the quantity of food was increased; his whole condition became more favorable, and he was finally sent home on the 20th of September, his term of service having expired. He was able to travel safely, and his wounds were almost closed.

Case XII. Wm. Hutchinson, age 32, Co. F, 107th New York, was struck by a bullet at Chancellorsville, May 3d, 1863, in the middle of the right tibia, which comminuted the bone, without, however, fracturing it through. He was admitted June 17th; the wound was discharging freely, and, shortly after his admission, several fragments of bone were removed. He was kept quiet in bed and his leg dressed with water. On the 10th of August, the open wound lost its healthy look, and the discharge became thin and offensive. On the 18th, the case was beyond doubt a mild one of hospital gangrene. There were the burning pain, the livid areola, the thickened everted edges, and the deep, tenacious, gray slough occupying the former seat of the opening. No effort was made to remove this necrosed tissue, and hence the application of the pure bromine produced less than the usual effect. For six days after the 18th, the bromine was applied daily, and followed by yeast poultices. No ulceration occurred after the first application; but at the expiration of the time stated, a florid, healthy surface replaced the gangrenous one; the excavation was deep and extensive enough to contain a large egg. After this complication the wound filled up rapidly, the man received a furlough, but was enfeebled by the long-continued discharge resulting from local necrosis.

Case XIII. James Hogan, age 47, private Co. A, 127th Pa. Vols., was struck at Fredericksburg, Va., December 13th, 1862, by a minie ball, which comminuted his right tibia. A resection of six inches of the shaft was performed at a field hospital. The fibula was uninjured. A tedious and almost hopeless convalescence, and the removal of sequestra from both upper and lower extremities of the tibia, found him, after the closure of the extensive incision, in good health, and with only a very small granulating surface near the tubercle of the tibia unhealed. This was attacked by hospital gangrene, was treated ineffectually with nitric acid, and penetrated so deeply as to make the involvement of the knee-joint probable.

Two or three applications of pure bromine at once checked the destructive action, but not until an opening large enough to receive a hen's egg had been produced. The granulation was rapid; the constitutional symptoms of gangrene soon disappeared, and the man recovered with a perfectly useless leg, since a hiatus of eight inches remained between the two extremities of the tibia. He was sent home on Oct. 12th, 1863, in good health, his term of service having expired.

*Douglas Hospital, Wdshington, D. C.*

